# RANdom SAmple Consensus (RANSAC) algorithm for material-informatics: application to photovoltaic solar cells

**DOI:** 10.1186/s13321-017-0224-0

**Published:** 2017-06-06

**Authors:** Omer Kaspi, Abraham Yosipof, Hanoch Senderowitz

**Affiliations:** 1 Department of Systems Engineering, Afeka – Tel-Aviv Academic College of Engineering, Tel-Aviv, Israel; 2 Department of Chemistry, Bar-Ilan University, 5290002 Ramat-Gan, Israel; 3 Faculty of Business Administration, College of Law & Business, 26 Ben Gurion Street, Ramat-Gan, P.O. Box 852, 5110801 Bnei Brak, Israel

**Keywords:** RANSAC, Material-informatics, QSAR, Photovoltaics, Solar Cells

## Abstract

An important aspect of chemoinformatics and material-informatics is the usage of machine learning algorithms to build Quantitative Structure Activity Relationship (QSAR) models. The RANdom SAmple Consensus (RANSAC) algorithm is a predictive modeling tool widely used in the image processing field for cleaning datasets from noise. RANSAC could be used as a “one stop shop” algorithm for developing and validating QSAR models, performing outlier removal, descriptors selection, model development and predictions for test set samples using applicability domain. For “future” predictions (i.e., for samples not included in the original test set) RANSAC provides a statistical estimate for the probability of obtaining reliable predictions, i.e., predictions within a pre-defined number of standard deviations from the true values. In this work we describe the first application of RNASAC in material informatics, focusing on the analysis of solar cells. We demonstrate that for three datasets representing different metal oxide (MO) based solar cell libraries RANSAC-derived models select descriptors previously shown to correlate with key photovoltaic properties and lead to good predictive statistics for these properties. These models were subsequently used to predict the properties of virtual solar cells libraries highlighting interesting dependencies of PV properties on MO compositions.

## Background

Material informatics is a rapidly developing field engaged with the application of informatics principles to materials science in order to assist in the discovery and development of new materials [1–5]. Developments in material informatics take advantage of the vast empirical and computational information on structures and properties of materials available in multiple databases such as MatWeb (http://www.matweb.com/) which includes properties for over 115,000 materials and MatDat (https://www.matdat.com/) which includes over 1000 datasets of materials, to name but a few. [6–10] Turning this large volume of information into knowledge could be performed in multiple ways using multiple data mining procedures. As an example, AFLOW [6] (http://aflowlib.org/) is a database of density functional theory (DFT) calculations performed on more than 1.5 million materials with known crystal structures. Isayev et al. [5]. used this database to introduce the term “material cartography” for representing a library of materials as a network. The resulting network was subsequently mined using various machine learning methods in search for materials with interesting properties.

A pre-requisite to any data mining procedure is a data curation stage [11]. Data curation is important for two main reasons: (1) Publically available data sets may contain multiple errors; (2) even a small number of errors may compromise the quality of QSAR models [11]. For example, Olah et al. [12, 13] have shown an error rate as high as 8% in the WOMBAT database and Young et al. [14] have recorded error rates between 0.1 and 3.4% in a variety of databases. More recently, Isayev et al. [5] have demonstrated several errors in the AFLOW database including duplicate compounds and incorrect extraction of literature data. In general, data curation involves steps like the removal of duplicates, compounds with wrong Lewis structures, compounds for which descriptors could not be calculated, and in case of experimentally measured data the removal of compounds which suffer from errors caused by the measurement process.

Due to the sheer size of material databases, data curation cannot be performed manually but rather requires a computational workflow. Indeed several such workflows have been reported in the literature [11, 15, 16]. However, even a stringent curation workflow cannot clean a database from noise that often accompanies experimental data. The presence of noise might mask the information that the data hold, thereby compromising data interpretation, model generation and decisions making.

In general, noise could be classified as either internal or external. Internal noise is inherent to the measurement process of the data, affects all data points, and is assumed to be distributed normally. In contrast, external noise results from sources exterior to the system due to an error in the measurement itself or from extreme behavior that does not match the overall behavior of the majority of samples. While all samples experience internal noise, some may also experience (greater) external noise and could therefore be regarded as outlier samples. Thus, an outlier is an observation on the dataset, which appears to be inconsistent with the rest of the data [17].

Important aspects of data mining in material informatics are database searching, similarity searches, and the usage of machine learning algorithms for pattern recognition and derivation of predictive models [18, 19]. Multiple terms have been used to describe such models including Quantitative Structure Activity/Property Relationship (QSAR/QSPR) models [20, 21], Quantitative Materials Structure–Property Relationships (QMSPR) models [5], and Quantitative Nanostructure Activity Relationship (QNAR) models [22]. All models attempt to correlate specific activities (or properties) for a set of materials with (calculated or measured) molecular descriptors by means of a mathematical model. Such models should both provide scientific insight into the problem in hand as well as allow for the prediction of the results of future experiments. An important characteristic of QSAR models is therefore their predictive power. However the presence of outliers (i.e., noise) may bias the dataset to the point of compromising the ability of machine learning algorithms to build predictive models. Consequently, a common practice of QSAR modeling is the prior removal of outlying samples prior to model generation [23]. Accordingly, several methods for the removal of outliers were reported in the literature [24–29].

Two more aspects of machine learning algorithms which critically affect performances are the selection of specific descriptors that best correlate with the activity under study from the initial pool of descriptors and the definition (and application) of the model’s applicability domain, namely, the region in material space in which the model is expected to give accurate predictions. Multiple descriptors selection (i.e. feature selection) methods have been developed including filter methods, wrapper methods and embedded methods [30]. Similarly several algorithms for the definition of applicability domains have been reported [31].

Most QSAR studies treat the removal of outliers, the selection of descriptors and the definition of applicability domain as separate stages within a QSAR workflow, often using different tools for each task [11, 20, 32, 33]. Thus, there is an interest in presenting a “one stop shop” algorithm for the performance of all tasks. The advantages of such an algorithm are the potential prevention of errors resulting from interfaces between different components as well as easier accessibility, in particular by non-experts. In contrast a “one stop shop” algorithm is by its nature non-modular, offering minimal flexibility in the modeling process.

With this in mind we present in this work the adaptation, implementation, and the first application of the RANdom SAmple Consensus (RANSAC) method [34] to the field of material-informatics by deriving predictive models for key photovoltaic properties of solar cells. RANSAC is a modeling tool widely used in the Image Processing field [34–36] primarily for image noise filtration. The algorithm produces and validates a linear QSAR model based on the Minimum Least Square (LMS) method by (1) filtering noisy samples (i.e., outliers), (2) selecting the best features (i.e., descriptors), (3) deriving a QSAR model from training set samples and (4) predicting the activity of test set samples while invoking the concept of applicability domain, all in a single process without the need of complementary processes. For prediction of samples not in the original test set (i.e., samples for which no activity data are available), RANSAC provides a statistical estimate for the probability of obtaining reliable predictions, i.e., predictions within a pre-defined number of standard deviations from the true values. These characteristics make RANSAC an appealing addition to the arsenal of tools available for the derivation of predictive QSAR models.

As a first application, we chose to test the performances of RANSAC in the important field of solar cells which emerge as one of the main resources for clean energy. Briefly, a typical solar cell (photovoltaic device) operates by: (1) Generation of charge carriers (electrons and holes) following the absorption of photons; (2) Separation of the photo-generated charge carriers via charge selective contact(s); (3) Collection of the photo-generated charge carriers at an external circuit resulting in electricity.

In particular we focus our attention on solar cells entirely composed of metal oxides (MOs). Such cells possess many favorable properties including natural abundance of the constituting materials, ease of fabrication and long time stability. However, such cells do not demonstrate sufficient efficiency in converting sunlight to electricity thereby requiring the development of new cells potentially composed of new MOs or MO combinations [37]. Such developments could be facilitated by the development of QSAR models to predict key solar cells properties such as current, voltage, and quantum efficiency. Yet despite their importance only few QSAR studies were reported on solar cells [38–40] and even fewer on MO-based solar cells [41].

MO-based solar cells are often produced using combinatorial techniques resulting in solar cell libraries [37, 42]. Following fabrication, the libraries are subjected to medium throughput measurements to characterize their composition/structure as well as their photovoltaic (PV) properties. Due to the technical challenges involved in both fabrication and characterization, the resulting libraries often contain noisy data [42] making them ideal candidates for the RANSAC algorithm. The main objective of the present study is therefore to establish the usefulness of the RANSAC algorithm in cleaning and analyzing datasets of solar cells libraries and predicting their PV properties. For this purpose, we used three recently published datasets experimentally-derived from two different solar cells libraries. The first library is a $TiO_{2} |Cu_{2} O$ library reported by Pavan et al. [43]. The library consists of two datasets, one with Ag back contacts and the other with Ag|Cu back contacts. The second library is a $TiO_{2} \left| {Co_{3} O_{4} } \right|MoO_{3}$ library reported by Majhi et al. [15]. The two libraries comprised of $TiO_{2} |Cu_{2} O$ based solar cells were previously modeled using *k* nearest neighbors (*kNN*) and genetic algorithm allowing for a facile comparison between the performances of the different algorithms. The third library ($TiO_{2} \left| {Co_{3} O_{4} } \right|MoO_{3} )$ was previously analyzed using visualization methods [36]. We demonstrate that the RANSAC algorithm filters the sample space from noisy data (i.e., outliers), automatically selects descriptors previous shown to correlate with key PV properties and generates models with good predictive statistics for these properties.

## Methods

### RANSAC overview

**Fig. 1:**
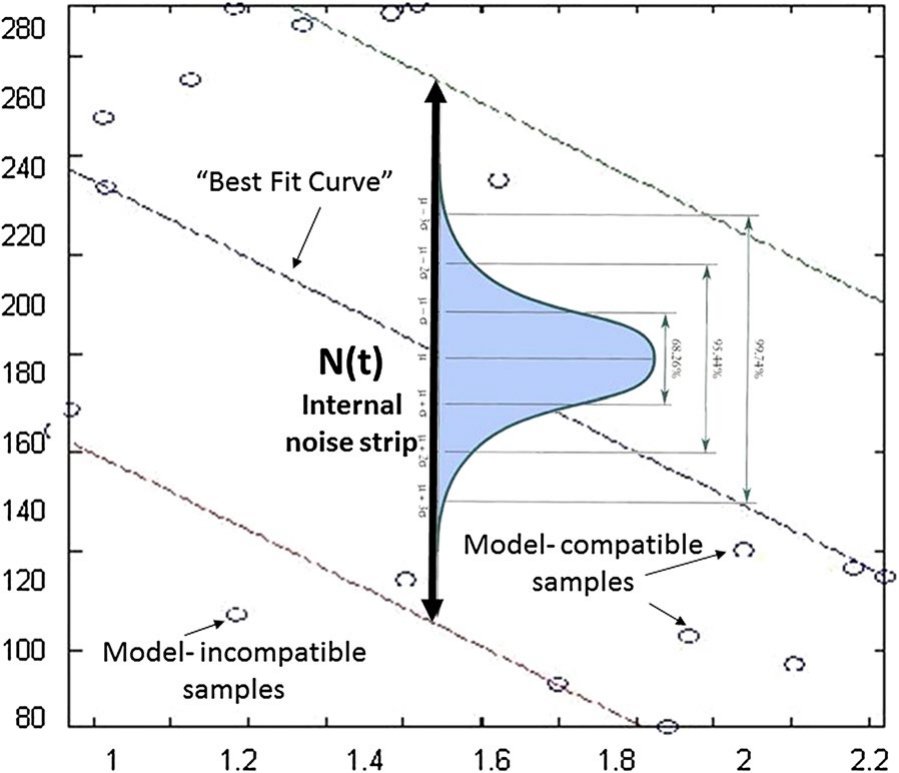
A possible RANSAC output where the desired model is of the first power (i.e., *straight line*). The algorithm assumes that due to internal noise, samples will not be exactly on the model but within a normal distribution around it. Conceptually, this variance forms a “strip” where all samples that lay within its boundaries are influenced by internal noise only. Samples within a “strip” are defined as model-compatible. Samples outside the “strip” are defined as model-incompatible

RANdom SAmple Consensus (RANSAC) [34] is a method for deriving a model based on linear regression, performed on input data that may include noisy samples (both internal and external noise). The basic assumption of the algorithm is that the measured activity ($Y_{measured} (\bar{x}))$ depends on a set of noise-free variables (e.g., descriptors; $\bar{x}$) and on noise added to them; Eq. (1).

1 $$Y_{measured} \left( {\bar{x}} \right) = Y_{{noise{-}free}} \left( {\bar{x}} \right) + N$$

where $Y_{{noise{-}free}} (\bar{x})$ is the expected activity in a noise-free environment and $N$ is a random internal noise. RANSAC assumes that the *internal* noise obeys the homoscedastic assumption, namely, that it has a constant distribution across all activity values. Using this assumption, boundaries could be set to form a “strip” that classifies the samples as either affected by internal noise only (model-compatible samples residing within the “strip”) or such that are affected both by internal and by external noise (model-incompatible samples residing outside the “strip”). Importantly, these boundaries should be a priori provided to the algorithm, based on the system’s characteristics and are expressed as the distance, in number of standard deviations (*n*), from the model (see below and Fig. 1).

**Fig. 2:**
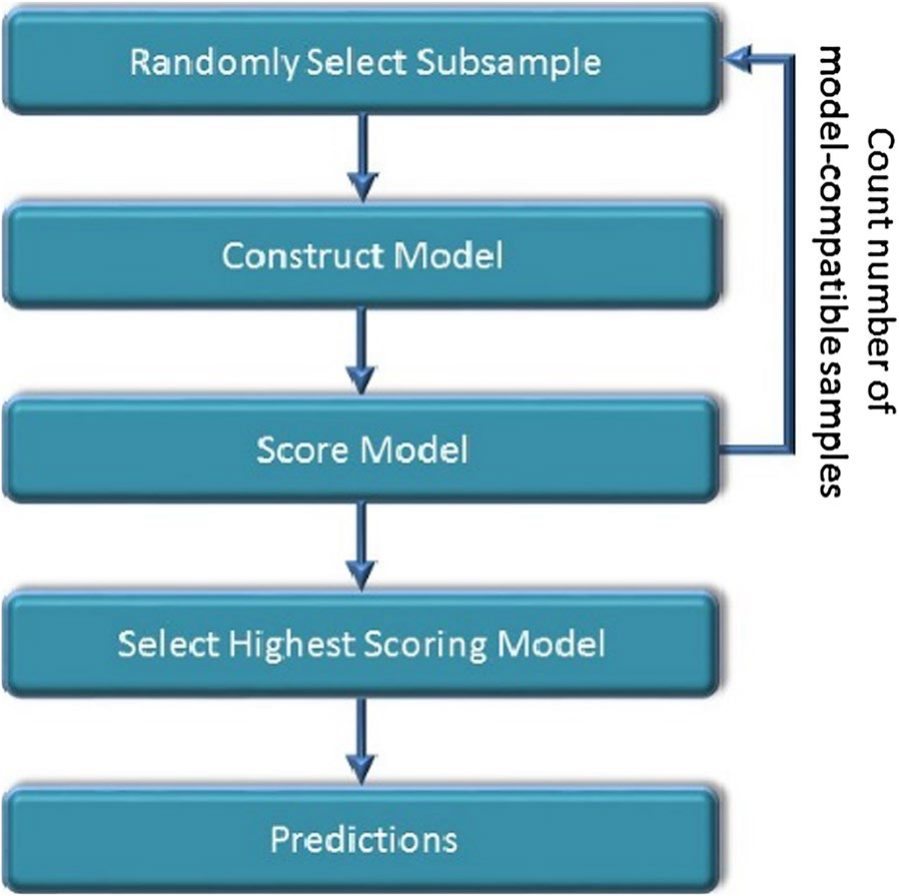
Description of the RANSAC algorithm as used for model construction

Mathematically, the following definition applies [Eq. (2)]:

2 $$\begin{array}{*{20}l} {if\,\frac{{Y_{measured} \left( {\bar{x}} \right) - Y_{calculated} \left( {\bar{x}} \right)}}{\sigma } > n} \hfill & {Model{-}Incompatible\,Sample} \hfill \\ {else} \hfill & {Model{-}Compatible\,Sample} \hfill \\ \end{array}$$

where $Y_{calculated} \left( {\bar{x}} \right)$ is the calculated activity (see below), σ is the standard deviation of the sample and *n* is the width of the “strip” (in units of σ). Operationally, RANSAC incorporates the following stages (Fig. 2): (1) *Model construction*: randomly select a subsample from the dataset and fit to it a linear curve using linear regressions Least Mean Squares (LMS). (2) *Model scoring*: classify all samples as either model-compatible or model incompatible (based on the a priori provided “strip” width). (3) *Iterative phase*: repeat steps *(1)* and *(2)* to build multiple models each based on other randomly selected subsamples. For each model count the number of model-compatible and model-incompatible samples (4) *Model selection*: select the model with the largest number of model-compatible samples, calculate LMS, discard model-incompatible samples (i.e., outliers) and calculate LMS again. This model will be used for subsequent predictions.

#### Model construction

For RANSAC to build a model, it must first draw a subsample from all the samples used for model training (i.e., training set) and use it to construct a regression line. For a single observation the model takes the form of Eq. (3):

3 $${\text{y}}_{\text{i}} = w_{0} x_{i0} + {\text{w}}_{1} x_{i1} + {\text{w}}_{2} x_{i1}^{2} + \cdots + {\text{w}}_{\text{p}} x_{i1}^{p} + {\text{w}}_{{{\text{p}} + 1}} x_{i2} + {\text{w}}_{{{\text{p}} + 2}} x_{i2}^{2} + \cdots + w_{p *d} x_{id}^{p}$$

Where *y* is the dependent variable, $\bar{x}$ is the vector of the independent variables (i.e., descriptors), *i* denotes sample *i*, *p* is the power of the best fit curve, *d* is the dimensionality of the model (i.e., number of descriptors) and $\bar{W}$ is a vector holding the weights calculated using the linear regression. Note that $\bar{W}$ may have zero values for one or more input descriptors meaning, that these descriptors were not selected by the model. For multiple samples, the matrix form is used [Eq. (4)]:

4 $$\bar{Y}_{calculated} = X\bar{W}$$

where

$$X = \left( {\begin{array}{*{20}c} {x_{10} } & \cdots & {x_{1d}^{p} } \\ \vdots & \ddots & \vdots \\ {x_{i0} } & \cdots & {x_{id}^{p} } \\ \end{array} } \right),\quad \bar{W} = \left[ {\begin{array}{*{20}c} {w_{0} } \\ \vdots \\ {w_{d *p} } \\ \end{array} } \right],\quad {\bar{\text{Y}}}_{\text{calculated}} = \left[ {\begin{array}{*{20}c} {y_{0} } \\ \vdots \\ {y_{d} } \\ \end{array} } \right]$$

5 $$\bar{W} = \left( {X^{T} X} \right)^{ - 1} X^{T} \bar{Y}$$

The size of the subsample drawn by RANSAC should match the power (*p*) of the desired equation [Eq. (3)]. For example, for an equation with *p* = 3, a subsample of size 4 should be drawn.

#### Model scoring

The basic assumption underlying the RANSAC algorithm is that the set of samples (expressed as data points) could be approximated by a model of a certain dimensionality (*d*), where each dimension is represented by a descriptor raised up to a maximum power (*p*) allowed for the model. If this assumption holds true, then one would expect to have most dataset points residing within a “strip” of a given width around the best fit curve calculated for a subsample (i.e., model compatible samples). The “strip” could be used for several purposes: (1) Scoring models by counting the number of dataset points residing within their boundaries (the larger the number, the better the model). Models are scored based on the entire training set and not only on the drawn sub-sample used for their construction. (2) Identifying outliers by observing training set samples residing outside the “strip’s” boundaries. (3) Defining the “strip” as the model’s applicability domain for test set predictions. RANSAC scores a model based on the number of model-compatible samples from within the training set.

#### Iterative phase and select highest scoring model

RANSAC is an iterative algorithm that requires many repetitions of the model construction and scoring phases (i.e., iterations) in order to obtain the best model. Furthermore, the number of the required iterations depends on the size of the dataset with larger datasets requiring more iterations. At each iteration, the algorithm counts the number of model-compatible samples and outputs the weights vector ($\bar{W}$) that corresponds to the highest ranked model (i.e., model with the highest score). For this model the LMS error is calculated both before and after the removal of outliers (i.e., model-incompatible samples). It is important to note that the size of the “strip” (which ultimately determines the number of model compatible samples) may vary between libraries and should be specifically chosen for each library.

#### Predictions

The best model emerging from the iterative phase is used for predictions. For test set samples, their known activities allow to classify them as either within or outside the model’s applicability domain (i.e., either within or outside the “strip”). The percentage of within-“strip” samples provides an estimate for the percentage of “correct” (i.e., within the predefined number of standard deviations (*n*) from the true value) predictions for “future” samples, that is samples with unknown activities. RANSAC does not feature an inherent applicability domain for individual samples although a descriptors based applicability domain approach could of course be used [31].

For all RANSAC’s applications described in this work the following parameters were used: The number of iterations was set to 10^5^ to derive a polynomial equation of the 5th power. The size of the “strip” (i.e. the models’ boundaries) was set to be ±1 standard deviation around $\bar{Y}_{measured}$ derived from the training set. The algorithm was coded in MATLAB version R2014a.

### Datasets

#### Metal-oxide solar cells library

**Fig. 3:**
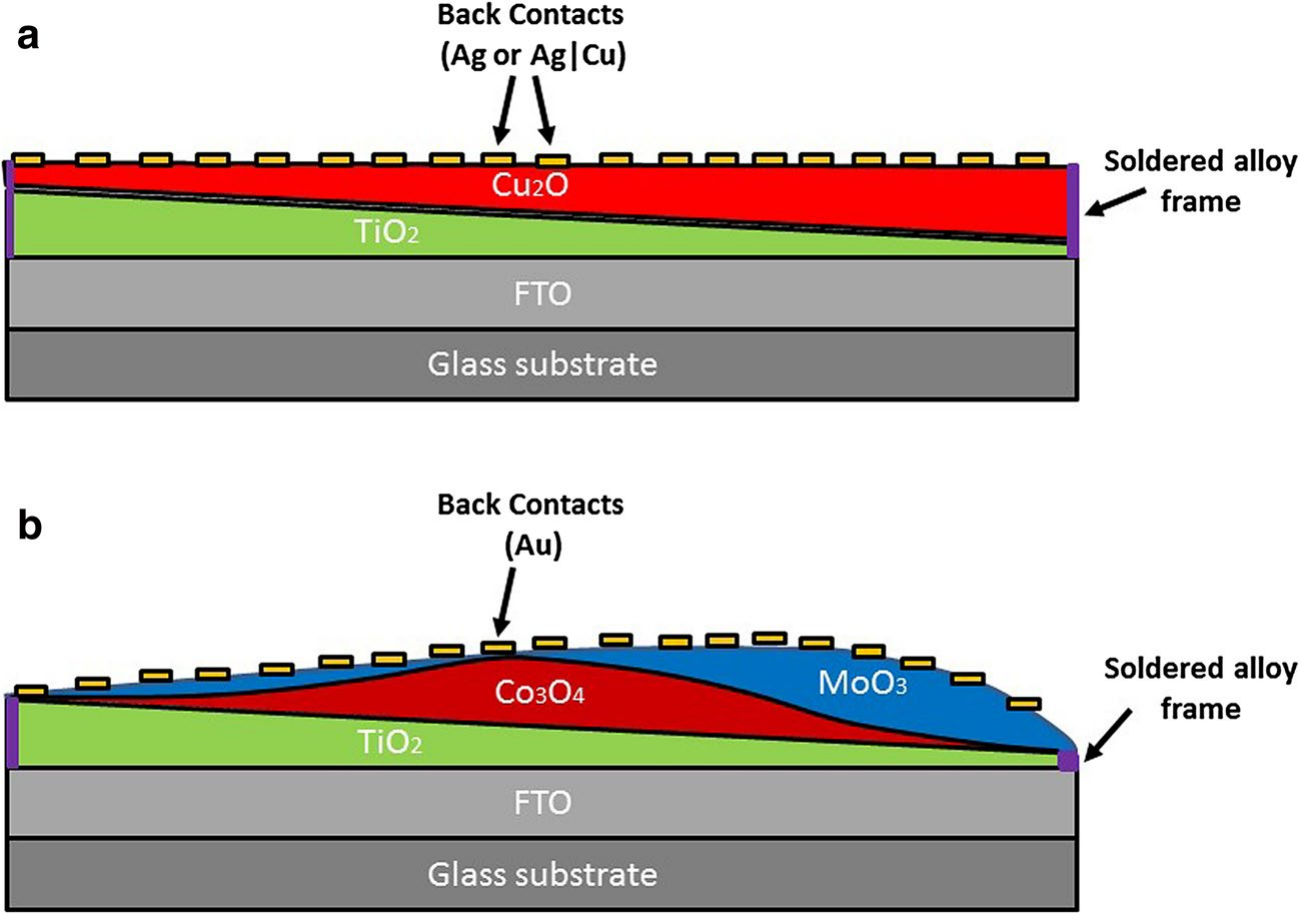
A schematic representation of the PV solar cells libraries. **a** $TiO_{2} |Cu_{2} O$ library (with Ag and Ag|Cu back contacts), **b** $TiO_{2} \left| {Co_{3} O_{4} } \right|MoO_{3}$ library

The basic assembly of MO solar cell library includes (see Fig. 3): (1) a transparent conducting oxide (TCO) coated on a glass, typically in the form of fluorine doped tin oxide (FTO); (2) a window layer, which is a wide band-gap n-type semiconductor (typically TiO_2_); (3) a light absorbing layer (absorber); (4) Metal back contact; (5) Metal frame (front contact) soldered directly onto the FTO.

#### $TiO_{2} |Cu_{2} O$ library (Fig. 3a)

An experimental library of solar cells was obtained from Pavan et al. [43]. This library was generated on precut glass coated with fluorine doped tin oxide (FTO) substrates onto which a TiO_2_ window layer with a linear gradient was deposited, followed by an absorber layer of Cu_2_O. Inserting two different grids of 13 × 13 = 169 back-contacts, namely, silver only (Ag) and silver and copper (Ag|Cu) deposited one after the other, lead to two sub-libraries (datasets) each consisting of 169 cells. In this work we omitted the non-photovoltaic cells leaving a total of 162 and 166 cells for the Ag and Ag|Cu back contact data base respectively.

#### $TiO_{2} \left| {Co_{3} O_{4} } \right|MoO_{3}$ library (Fig. 3b)

This library was constructed in a manner roughly similar to the $TiO_{2} |Cu_{2} O$ libraries, with the same window layer (TiO_2_) but different target metal oxide for the absorber layer (Co_3_O_4_) and also included a third recombination layer (MoO_3_). On top of the MoO_3_ layer a 13 × 13 grid of Au back contacts was placed, thus forming a library of 169 cells. The library was characterized by the varying thicknesses of the TiO_2_, Co_3_O_4_, MoO_3_ layers. For this library 19 cells were removed due to lack of photovoltaic activities (thus 150 cells remained).

#### Library characterization

Each solar cell was characterized by its material descriptors (independent variables) and experimentally measured photovoltaic activities (dependent variables). Material descriptors included the thickness of the window layer ($d_{{TiO_{2} }}$), the thickness of the absorber layers ($d_{{Cu_{2} o}} \,{\text{and}}\,d_{{Co_{3} O_{4} }}$), the thickness of the recombination layer ($d_{{MoO_{3} }}$), the thickness ratio between the absorber layer and the sum of the absorber and window layers (*ratio*), the thickness ratio between the absorber layer and the sum of the absorber and the recombination layers (*ratio_AR*), and the band gap of absorber layer (*BGP*). The band gap is the energy difference (in electron volts) between the top of the valence band and the bottom of the conduction band. Overall, for the $TiO_{2} |Cu_{2} O$ libraries four descriptors were consider namely: $d_{{TiO_{2} }} , d_{{Cu_{2} o}} ,ratio,$ and *BGP*, and for the $TiO_{2} \left| {Co_{3} O_{4} } \right|MoO_{3}$ library five descriptors were consider namely: $d_{{TiO_{2} }} , d_{{Co_{3} O_{4} }} , d_{{MoO_{3} }} ,$ $ratio,$ and $ratio\_AR$. Tables 1 and 2 present the range values for each of the descriptors.

**Table 1:** Descriptor ranges for the $TiO_{2} |Cu_{2} O$ library (with Ag and Ag|Cu back contacts)

	$TiO_{2} |Cu_{2} O$ (both libraries)
$d_{{TiO_{2} }}$ (nm)	70.0–311.5
$d_{{Cu_{2} o}}$ (nm)	249.0–596.0
Ratio	0.51–0.89
BGP (eV)	0.21–2.50

**Table 2:** Descriptor ranges for the $TiO_{2} \left| {Co_{3} O_{4} } \right|MoO_{3}$ library

	$$TiO_{2} \left| {Co_{3} O_{4} } \right|MoO_{3}$$
$d_{{TiO_{2} }}$ (nm)	259.0–355.0
$d_{{Co_{3} O_{4} }}$ (nm)	30.7–245.0
$d_{{MoO_{3} }}$ (nm)	38.9–61.8
Ratio	0.08–0.43
Ratio_AR	0.38–0.86

**Fig. 4:**
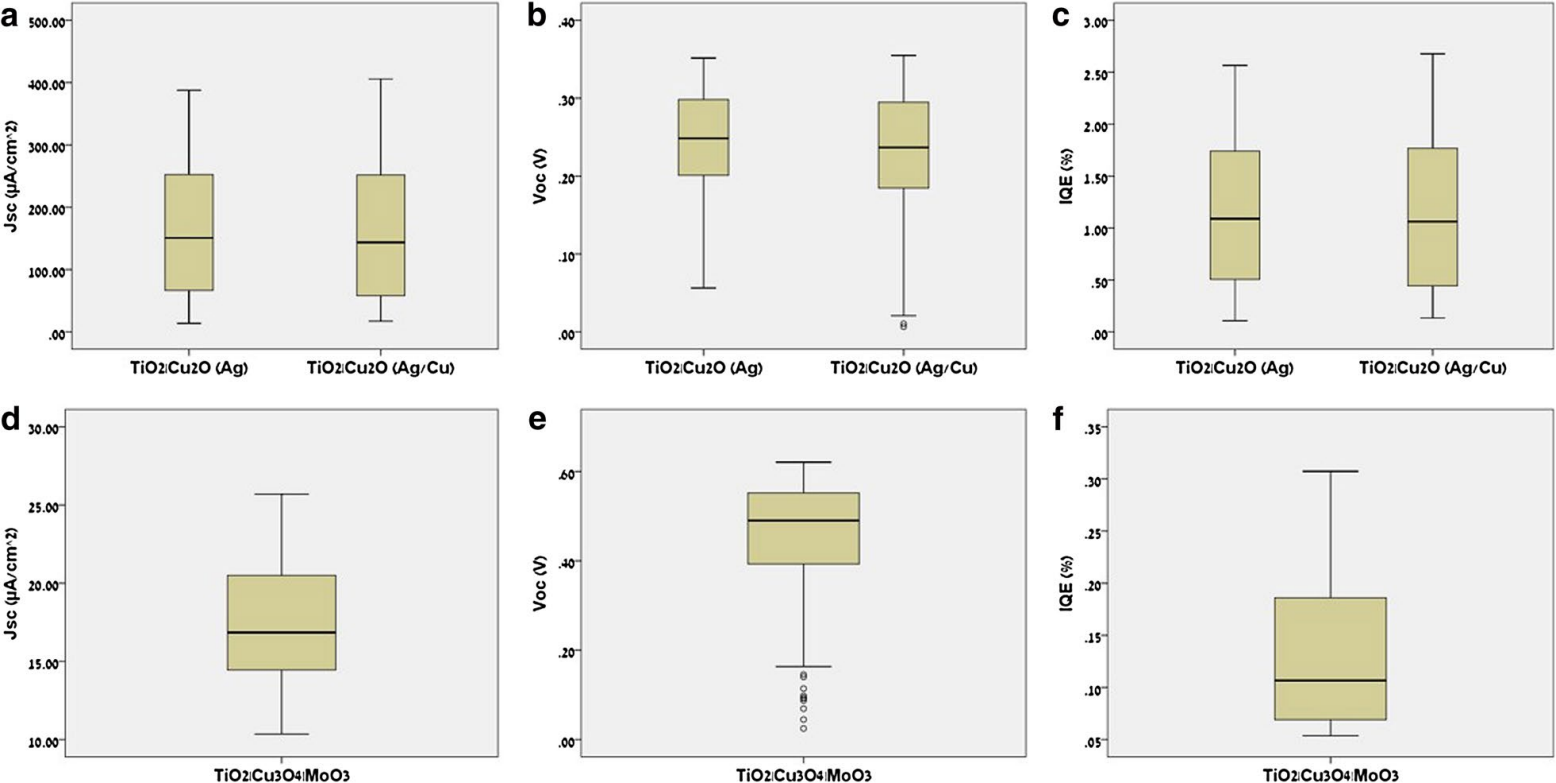
*Boxplots* of the three PV activities (*J*_*SC*_ *, Voc*, and *IQE*). **a**–**c** The three PV activities distribution for the $TiO_{2} |Cu_{2} O$ library (with Ag and Ag|Cu back contacts). **d**–**f** The three PV activities distribution for the $TiO_{2} \left| {Co_{3} O_{4} } \right|MoO_{3}$ library. The *boxplots* show the median values (*solid horizontal line*), 50th percentile values (*box outline*), the lower and upper quartile (*whiskers*, *vertical lines*), and outlier values (*open circles*)

In this work we focused on three experimentally measured PV activities (dependent variables, end points): (1) the short circuit photocurrent density (*J*_*SC*_) which is the current density through the solar cell when the voltage across the cell is zero. (2) The open circuit voltage (*Voc*) which is the maximum voltage available from a solar cell. This voltage occurs at an open circuit. (3) The internal quantum efficiency (*IQE*) which reflects the charge separation and collection efficiencies of a device and is calculated by Eq. (6) where $J_{max}$ is the maximum theoretical calculated photocurrent. The distributions of the three PV activities are represented by boxplots in Fig. 4 and their ranges are given in Table 3.

**Table 3:** Activity ranges for the three libraries

	$TiO_{2} |Cu_{2} O$ (Ag)	$TiO_{2} |Cu_{2} O$ (Ag|Cu)	$TiO_{2} \left| {Co_{3} O_{4} } \right|MoO_{3}$
*J*_*SC*_ $\left( {\upmu{\text{A/cm}}^{2} } \right)$	13.9–387.8	17.21–405.86	10.35–25.7
*V*_*OC*_ $\left( {\text{V}} \right)$	0.06–0.35	0.01–0.35	0.03–0.62
*IQE* (%)	0.11–2.56	0.13–2.68	0.05–0.31

6 $$IQE = \frac{Jsc}{{J_{max} }}$$

#### Model fitting and statistical parameters

The datasets were divided into training and validation (test) sets using a recently published representativeness algorithm [44]. Subsets selected by this algorithm were previously employed as external validation sets in QSAR modeling [24, 25, 41, 44]. Each dataset was divided into a training set composed of 80% of the original dataset (130, 134 and 120 cells for the $TiO_{2} |Cu_{2} O$ with Ag back contacts, $TiO_{2} |Cu_{2} O$ with Ag|Cu back contacts and $TiO_{2} \left| {Co_{3} O_{4} } \right|MoO_{3}$ datasets, respectively) and a test set containing the remaining cells (32 samples for the $TiO_{2} |Cu_{2} O$ with Ag and Ag|Cu back contact and 30 samples for the $TiO_{2} \left| {Co_{3} O_{4} } \right|MoO_{3}$ dataset). The $TiO_{2} |Cu_{2} O$ libraries with Ag and Ag|Cu Back Contacts were previously modeled by Yosipof et al. [41]. For the purpose of comparison, the training and test sets described above, were made identical to those described by Yosipof et al. [41].

To evaluate the RANSAC model performances on the training set we used $Q_{train}^{2}$ as expressed by Eq. (7). The RANSAC algorithm excludes samples from the training set-based error calculation if residing outside the model’s boundaries (e.g., “strip”). Thus the model’s error is derived without these samples. This is analogous to outlier removal. Below we therefore report two $Q_{train}^{2}$ estimates, the first based on all samples and the second based on samples surviving RANSAC’s inherent outlier removal.

The performances of the RANSAC algorithm on the test set ($Q_{ext}^{2}$) were calculated in a similar manner [Eq. (8)]. Similarly to outlier removal, the “strip” calculated by the RANSAC algorithm was used to evaluate the applicability domain (AD) of the resulting model. Accordingly, two estimates of $Q_{ext}^{2}$ were calculated one pertaining to the entire test set and one, for that portion of the test set which resided within the model’s applicability domain.

7 $$Q_{train}^{2} = 1 - \frac{{\mathop \sum \nolimits \left( {Y_{measured,train} - Y_{predicted,train} } \right)^{2} }}{{\mathop \sum \nolimits \left( {Y_{measured,train} - \bar{Y}_{measured,train} } \right)^{2} }}$$

8 $$Q_{ext}^{2} = 1 - \frac{{\mathop \sum \nolimits \left( {Y_{measured,test} - Y_{predicted, test} } \right)^{2} }}{{\mathop \sum \nolimits \left( {Y_{measured,test} - \bar{Y}_{measured,test} } \right)^{2} }}$$

where $Y_{measured}$ is the experimental result, $Y_{predicted}$ is the predicted value and $\bar{Y}_{measured}$ is the mean of the experimental results over training set samples.

In addition, we used the R^2^ (squared correlation coefficient) between the predicted ($Y_{predicted}$) and the experimental ($Y_{measured }$) data for both training and test set.

Finally, to assess model significance and to rule-out chance correlation, Y-randomization procedure was applied to all models.

## Result and discussion

### Performances of RANSAC-derived models

The RANSC algorithm was applied to the three datasets described above. For each dataset, three models were derived to describe their photovoltaic (PV) properties (*J*_*SC*_ *, V*_*OC*_ and *IQE*). Table 4 presents the number of training set and test set samples found to reside within the model’s “strip” (i.e., model-compatible samples). Model-incompatible samples in the training and test sets are referred to as outliers and outside of the model’s AD, respectively. As can clearly be seen, the vast majority (≥85%) of the samples are included within the “strip” for both the training and test sets. This suggests that (1) predictive models could likely be derived for this dataset and (2) the model described by the “strip” forming curve approximates most of the training set and test set samples to within one standard deviation (the pre-defined “strip” width; see Methods section) from their experimental values. One could therefore propose that the majority of future samples will be similarly predicted. However, in two cases the number of model compatible cells was below the 85% threshold (the *V*_*OC*_ models for the $TiO_{2} \left| {Co_{3} O_{4} } \right|MoO_{3}$ library with 84 and 80% of model compatible cells for the training and test sets, respectively), indicating higher variance for this property in this dataset in comparison with the other properties/datasets. In accord with this observation, the performances of the *V*_*OC*_ model from the $TiO_{2} \left| {Co_{3} O_{4} } \right|MoO_{3}$ library were exceptionally poor (Table 5). This model was therefore excluded from the analysis reported below.

**Table 4:** Number of model-compatible samples for the three datasets based on the RANSAC models

	J_SC_	V_OC_	IQE
$$TiO_{2} |Cu_{2} O$$ (Ag)			
# Model—compatible training samples	125/130 (96%)	120/130 (92%)	125/130 (96%)
# Model—compatible test samples	28/32 (88%)	32/32 (100%)	28/32 (88%)
$$TiO_{2} |Cu_{2} O$$ (Ag|Cu)			
# Model—compatible training samples	131/134 (98%)	127/134 (95%)	129/134 (96%)
# Model—compatible test samples	30/32 (94%)	30/32 (94%)	31/32 (97%)
$$TiO_{2} \left| {Co_{3} O_{4} } \right|MoO_{3}$$			
# Model—compatible training samples	118/120 (98%)	101/120 (84%)	120/120 (100%)
# Model—compatible test samples	30/30 (100%)	24/30 (80%)	30/30 (100%)

**Table 5:** RANSC model performance for the three datasets

library	Activity	$Q_{train}^{2} (R^{2} )$	$Q_{train}^{2}$ $(R^{2} )$ (no outliers)	$Q_{ext}^{2} (R^{2} )$	$Q_{ext}^{2}$ (AD) $(R^{2} )$
$TiO_{2} |Cu_{2} O$ (Ag)	*J*_*SC*_	0.75 (0.77)	0.82 (0.84)	0.69 (0.75)	0.87 (0.89)
	*V*_*OC*_	0.62 (0.63)	0.65 (0.66)	0.80 (0.80)	0.80 (0.80)
	*IQE*	0.71 (0.72)	0.79 (0.82)	0.69 (0.76)	0.83 (0.87)
$TiO_{2} |Cu_{2} O$ (Ag|Cu)	*J*_*SC*_	0.74 (0.78)	0.78 (0.82)	0.76 (0.81)	0.84 (0.88)
	*V*_*OC*_	0.57 (0.62)	0.73 (0.78)	0.62 (0.68)	0.73 (0.78)
	*IQE*	0.72 (0.74)	0.78 (0.81)	0.78 (0.82)	0.82 (0.86)
$TiO_{2} \left| {Co_{3} O_{4} } \right|MoO_{3}$	*J*_*SC*_	0.77 (0.78)	0.78 (0.79)	0.82 (0.83)	0.82 (0.83)
	*V*_*OC*_	−0.06 (0.03)	0.25 (0.36)	0.00 (0.10)	0.33 (0.31)
	*IQE*	0.85 (0.86)	0.85 (0.86)	0.79 (0.81)	0.79 (0.81)

**Table 6:** A comparison of model coverage, based on test set samples, between RANSAC and *k*NN models

Library	Activity	RANSAC coverage (%)	*k*NN coverage* (%)
$TiO_{2} |Cu_{2} O$ (Ag)	*J*_*SC*_	88	91
	*V*_*OC*_	100	84
	*IQE*	88	91
$TiO_{2} |Cu_{2} O$ (Ag|Cu)	*J*_*SC*_	94	79
	*V*_*OC*_	94	79
	*IQE*	97	73

^*^The data for *k*NN were taken from Table 5 in Ref. [41]

Overall, the RANSAC algorithm led to models with good statistical parameters (Table 5) for training set samples for *J*_*SC*_ ($Q_{train}^{2}$ between 0.74 and 0.77), *Voc* ($Q_{train}^{2}$ between 0.57 and 0.62 excluding the $TiO_{2} \left| {Co_{3} O_{4} } \right|MoO_{3}$ library; see above) and IQE ($Q_{train}^{2}$ between 0.71 and 0.85). Upon the removal of outliers, the statistical parameters for all models improved with the largest improvement being obtained for *V*_*OC*_ ($Q_{train}^{2}$ between 0.78–0.82, 0.65–0.73 (excluding the $TiO_{2} \left| {Co_{3} O_{4} } \right|MoO_{3}$) and 0.78–0.85 for *J*_*SC*_, *V*_*OC*_ and IQE, respectively).

The performances of the RANSAC models on the test set samples followed a trend similar to that observed for the training set. Thus, for all test sets, $Q_{ext}^{2}$ was found to be between 0.69–0.82, 0.62–0.80, and 0.69–0.79 for *J*_*SC*_, *V*_*OC*_ (excluding the $TiO_{2} \left| {Co_{3} O_{4} } \right|MoO_{3} library$) and IQE, respectively. Similar results were obtained for R^2^ values between the predicted and the actual activities (Table 5). As expected for datasets devoid of significant activity cliffs, when considering only samples within the models’ applicability domains, these numbers improved to 0.82–0.87 and 0.79–0.83 for *J*_*SC*_, and IQE, respectively. For *V*_*OC*_ of the $TiO_{2} |Cu_{2} O$ (Ag) library, no test set samples were filtered by the applicability domain leading to no change in model performances ($Q_{ext}^{2}$ = 0.80). However for this property a significant increase in the $TiO_{2} |Cu_{2} O$ (Ag|Cu) library upon the removal of only two samples was observed ($Q_{ext}^{2}$ = 0.62 and 0.73 without and with the model’s AD, respectively).

**Fig. 5:**
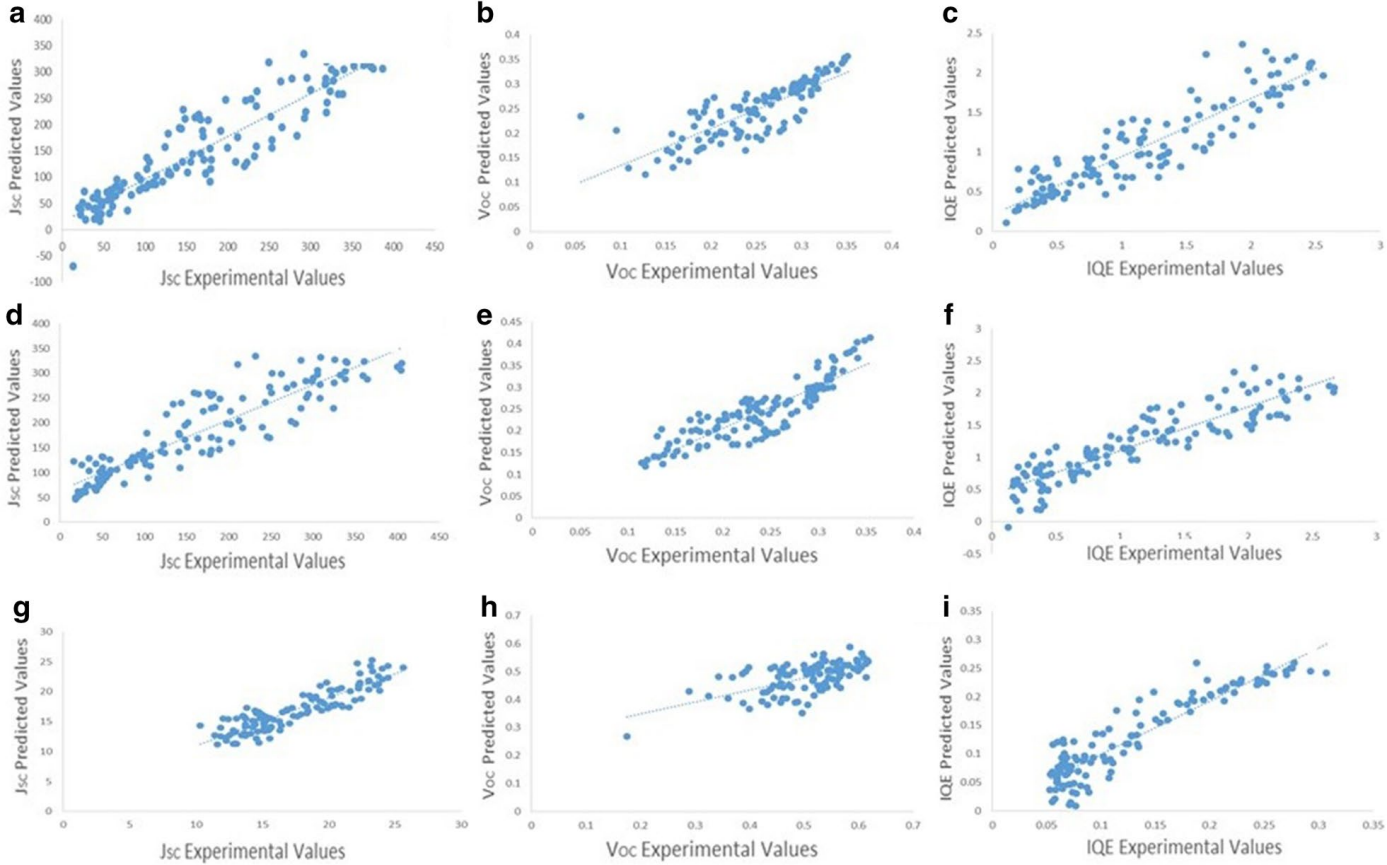
Predicted versus experimental PV properties for train set samples following the removal of outliers. **a**–**c** *J*_*SC*_, *V*_*OC*_ and *IQE* for the TiO_2_|Cu_2_O library with Ag back contacts, **d**–**f** *J*_*SC*_, *V*_*OC*_ and *IQE* for the TiO_2_|Cu_2_O library with Ag|Cu back contacts, **g**–**i** *J*_*SC*_, *V*_*OC*_ and *IQE* for the TiO_2_|Co_3_O_4_|MoO_3_ library

**Fig. 6:**
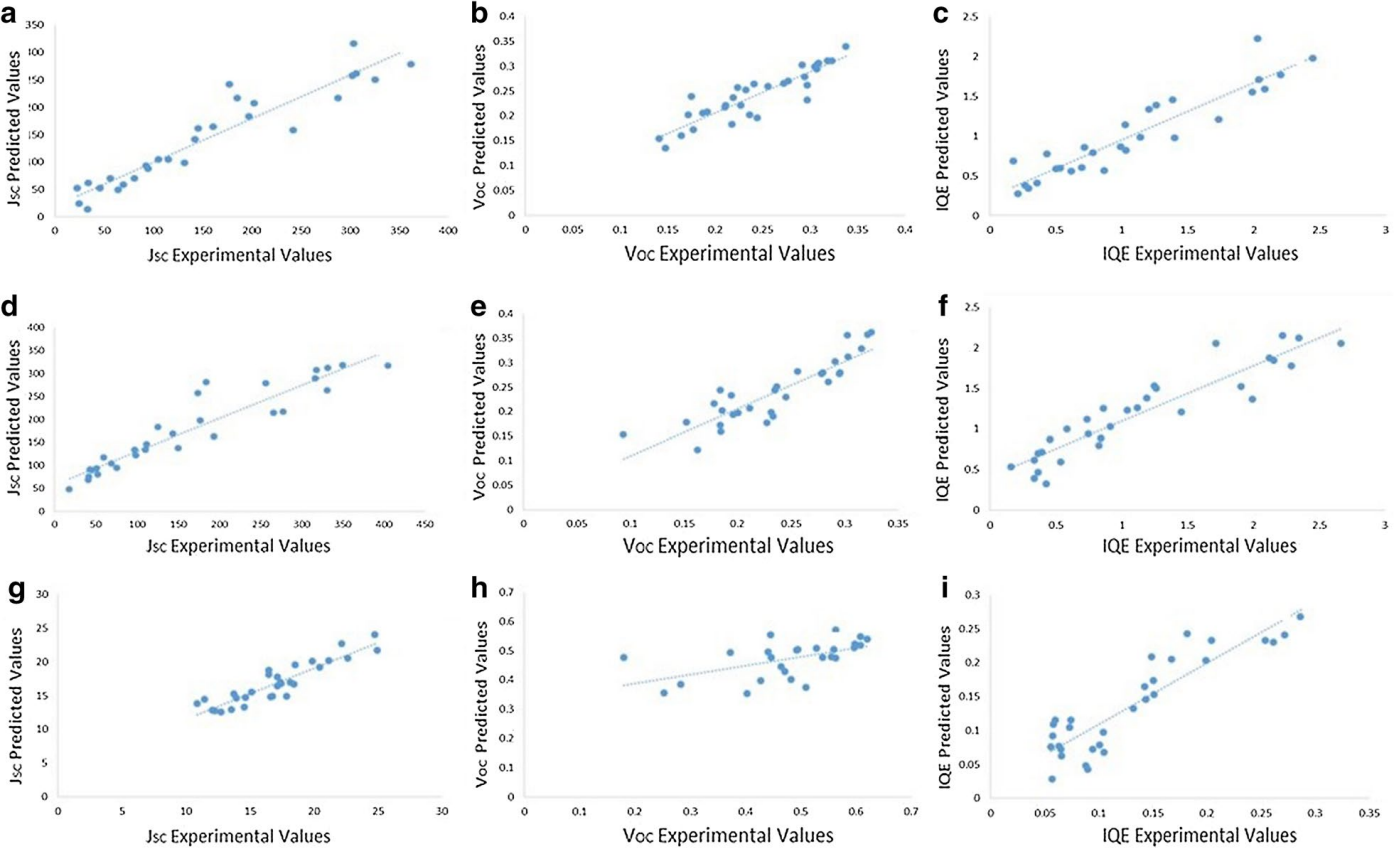
Predicted versus experimental PV properties for test set samples residing with the models applicability domains. **a**–**c** *J*_*SC*_, *V*_*OC*_ and *IQE* for the TiO_2_|Cu_2_O library with Ag back contacts, **d**–**f** *J*_*SC*_, *V*_*OC*_ and *IQE* for the TiO_2_|Cu_2_O library with Ag|Cu back contacts, **g**–**i** *J*_*SC*_, *V*_*OC*_ and *IQE* for the TiO_2_|Co_3_O_4_|MoO_3_ library

Figures 5 and 6 present predicted versus experimentally measured values for all three PV properties considered in this work across the three datasets following outlier removal for training set samples and considering only samples within the models applicability domains for the test set.

Finally, Y-randomization procedure was applied to all models and no statistically significant models were derived.

Two of the above described datasets [$TiO_{2} |Cu_{2} O$ (Ag) and $TiO_{2} |Cu_{2} O$ (Ag|Cu)] were previously modeled by Yosipof et al. [41] using *k*NN and a Genetic Programming (GP) approach, thereby allowing for a direct comparison between the performances of the resulting models (the results of *k*NN and GP models from Yosipof et al. [41] are presented in Table 7). GP produced models with $Q_{ext}^{2}$ values between 0.74–0.76, 0.50–0.78 and 0.72 for *J*_*SC*_, *V*_*OC*_ and IQE respectively. The corresponding numbers obtained by RANSAC are $Q_{ext}^{2}$ = 0.69–0.76, 0.62–0.80, and 0.69–0.78 for *J*_*SC*_, *V*_*OC*_ and IQE, respectively, with no AD and $Q_{ext}^{2}$ = 0.84–0.87, 0.73–0.80 and 0.82–0.83 for *J*_*SC*_, *V*_*OC*_ and IQE, respectively, with AD. These results suggest that the performances of the RANSAC models are similar to those of the GP with no consideration of the AD and provide significant improvement upon the application of AD. Of note, there is no inherent definition of AD in the GP method. For *k*NN, $Q_{ext}^{2}$ was reported to be 0.89–0.92, 0.56–0.89, and 0.87–0.91 for *J*_*SC*_, *V*_*OC*_ and IQE, respectively, with no AD and $Q_{ext}^{2}$ 0.88–0.92, 0.55–0.89 and 0.87–0.89 for *J*_*SC*_, *V*_*OC*_ and IQE, respectively, with AD. Thus, *k*NN provides models with higher prediction statistics than RANSAC in particular when the AD is not considered. However, the performances of RANSAC approach those of *k*NN upon the introduction of the AD. Moreover, the test set coverage provided by RANSAC is generally higher than that provided by *k*NN (Table 6). Finally, in contrast with *k*NN, RANSAC provides a model in the form of a QSAR equation which enhances model interpretability.

**Table 7:** *k*NN and GP models performance retrieved from Yosipof et al. [41]

Library	Activity	$Q_{train}^{2}$		$Q_{ext}^{2} (R^{2} )$	$Q_{ext}^{2}$ $(R^{2} )$ (AD)	$Q_{ext}^{2} (R^{2} )$
		*k*NN	GP	*k*NN	*k*NN	GP
$TiO_{2} |Cu_{2} O$ (Ag)	*J*_*SC*_	0.92	0.74	0.92 (0.92)	0.92 (0.92)	0.76 (0.76)
	*V*_*OC*_	0.78	0.65	0.89 (0.89)	0.89 (0.89)	0.78 (0.77)
	*IQE*	0.91	0.70	0.87 (0.87)	0.87 (0.87)	0.72 (0.73)
$TiO_{2} |Cu_{2} O$ (Ag|Cu)	*J*_*SC*_	0.92	0.76	0.89 (0.89)	0.88 (0.89)	0.74 (0.76)
	*V*_*OC*_	0.74	0.61	0.56 (0.55)	0.55 (0.54)	0.50 (0.50)
	*IQE*	0.9	0.72	0.91 (0.91)	0.89 (0.89)	0.72 (0.73)

### RANSAC as a feature selection tool

Table 8 presents the model equations produced by RANSAC for the different PV properties of the three datasets.

**Table 8:** RANSAC derived models for different PV properties

PV Property	Model
$TiO_{2} |Cu_{2} O$ (Ag)	$J_{SC} \,\left( {\upmu{\text{A/cm}}^{ 2} } \right) = - 7.9 \times 10^{ - 5} d_{{TiO_{2} }}^{3} + 6.32 \times 10^{ - 7} d_{{TiO_{2} }}^{4} - 1.3 \times 10^{ - 9} d_{{TiO_{2} }}^{5} + 7.27 \times 10^{ - 6} d_{{Cu_{2} o}}^{3} - 1.8 \times 10^{ - 8} d_{{Cu_{2} o}}^{4} + 1.47 \times 10^{ - 11} d_{{Cu_{2} o}}^{5}$
	$$V_{OC} \,\left( {\text{V}} \right) = 4.44 \times 10^{ - 8} d_{{TiO_{2} }}^{3} - 2 \times 10^{ - 10} d_{{TiO_{2} }}^{4} + 2.12 \times 10^{ - 13} d_{{TiO_{2} }}^{5} + 1.76 \times 10^{ - 8} d_{{Cu_{2} o}}^{3} - 5.7 \times 10^{ - 11} d_{{Cu_{2} o}}^{4} + 4.87 \times 10^{ - 14} d_{{Cu_{2} o}}^{5}$$
	$${\text{IQE}}\,\left( {\% } \right) = - 9.6 \times 10^{ - 8} d_{{TiO_{2} }}^{3} + 1.43 \times 10^{ - 9} d_{{TiO_{2} }}^{4} - 3.7 \times 10^{ - 12} d_{{TiO_{2} }}^{5} + 3.39 \times 10^{ - 8} d_{{Cu_{2} o}}^{3} - 9.2 \times 10^{ - 11} d_{{Cu_{2} o}}^{4} + 8.52 \times 10^{ - 14} d_{{Cu_{2} o}}^{5}$$
$TiO_{2} |Cu_{2} O$ (Ag|Cu)	$J_{SC} \,\left( {\upmu{\text{A/cm}}^{ 2} } \right) = 1.69 \times 10^{ - 5} d_{{TiO_{2} }}^{3} - 8.5 \times 10^{ - 8} d_{{TiO_{2} }}^{4} + 1.13 \times 10^{ - 10} d_{{TiO_{2} }}^{5} + 3.15 \times 10^{ - 6} d_{{Cu_{2} o}}^{3} - 1.9 \times 10^{ - 9} d_{{Cu_{2} o}}^{4} - 1.7 \times 10^{ - 12} d_{{Cu_{2} o}}^{5}$
	$$V_{OC} \,\left( {\text{V}} \right) = 9.31 \times 10^{ - 9} d_{{TiO_{2} }}^{3} + 4.01 \times 10^{ - 11} d_{{TiO_{2} }}^{4} - 2 \times 10^{ - 13} d_{{TiO_{2} }}^{5} + 2.04 \times 10^{ - 8} d_{{Cu_{2} o}}^{3} - 6.9 \times 10^{ - 11} d_{{Cu_{2} o}}^{4} + 6.18 \times 10^{ - 14} d_{{Cu_{2} o}}^{5}$$
	$${\text{IQE}}\,\left( {\% } \right) = - 9.47 \times 10^{ - 7} d_{{TiO_{2} }}^{3} + 7.2 \times 10^{ - 9} d_{{TiO_{2} }}^{4} - 1.4 \times 10^{ - 11} d_{{TiO_{2} }}^{5} + 1.2 \times 10^{ - 7} d_{{Cu_{2} o}}^{3} - 3.6 \times 10^{ - 10} d_{{Cu_{2} o}}^{4} + 3.08 \times 10^{ - 13} d_{{Cu_{2} o}}^{5}$$
$TiO_{2} \left| {Co_{3} O_{4} } \right|MoO_{3}$	$J_{SC} \,\left( {\upmu{\text{A/cm}}^{ 2} } \right) = - 1.5 \times 10^{ - 8} d_{{Co_{3} O_{4} }}^{4} + 6.09 \times 10^{ - 11} d_{{Co_{3} O_{4} }}^{5} + 3.74 \times 10^{ - 6} d_{{MoO_{3} }}^{4} - 4.5 \times 10^{ - 8} d_{{MoO_{3} }}^{5} + 5.17 \times 10^{ - 9} d_{{TiO_{2} }}^{4} - 1.3 \times 10^{ - 11} d_{{TiO_{2} }}^{5}$
	$$V_{OC} \,\left( {\text{V}} \right) = 2.08 \times 10^{ - 10} d_{{Co_{3} O_{4} }}^{4} - 6.8 \times 10^{ - 13} d_{{Co_{3} O_{4} }}^{5} + 4.42 \times 10^{ - 7} d_{{MoO_{3} }}^{4} - 6.7 \times 10^{ - 9} d_{{MoO_{3} }}^{5} - 4 \times 10^{ - 11} d_{{TiO_{2} }}^{4} + 7.35 \times 10^{ - 14} d_{{TiO_{2} }}^{5}$$
	$${\text{IQE}}\,\left( {\% } \right) = - 3.9 \times 10^{ - 7} d_{{Co_{3} O_{4} }}^{3} + 3.26 \times 10^{ - 9} d_{{Co_{3} O_{4} }}^{4} - 7.2 \times 10^{ - 12} d_{{Co_{3} O_{4} }}^{5} - 3.9 \times 10^{ - 11} d_{{MoO_{3} }}^{5} + 1.52 \times 10^{ - 10} d_{{TiO_{2} }}^{4} - 3.9 \times 10^{ - 13} d_{{TiO_{2} }}^{5}$$

For both $TiO_{2} |Cu_{2} O$ datasets it is evident that while four descriptors were evaluated by RANSAC, only two were picked by the algorithm as predictors of photovoltaic activities. Importantly, these two descriptors give rise to six terms in the resulting QSAR equations due to their power form. Thus, RANSAC “expands” the small number of final descriptors by using them in multiple forms. A potential drawback of the resulting models is therefore reduced interpretability of terms including “high power” descriptors. The $TiO_{2} \left| {Co_{3} O_{4} } \right|MoO_{3}$ dataset was characterized by five descriptors and only three were selected by RANSAC leading to models with six terms (Table 8). 

The features selected by the RANSAC algorithm could be compared with those selected by the *k*NN and GP models reported by Yosipof et al. [41]. As can be deduced from Table 9, all methods selected the same descriptors for the $TiO_{2} |Cu_{2} O$ (Ag) library while *k*NN replaced $d_{{TiO_{2} }}$ by the *ratio* descriptor for the $TiO_{2} |Cu_{2} O$ (Ag|Cu) library. While GP sometimes selected a smaller number of “base descriptors”, it compensated for this smaller number by incorporating these descriptors in more complex mathematical equations. In contrast, the RANSAC algorithm is limited to simple polynomial equation (to the 5th power in this study).

**Table 9:** Featured selected for the $TiO_{2} |Cu_{2} O$ libraries by the various methods

Library	Activity	RANSAC	GP	*k*NN
$TiO_{2} |Cu_{2} O$ (Ag)	*J*_*SC*_	$d_{{Cu_{2} o}} ,d_{{TiO_{2} }}$	$d_{{Cu_{2} o}}$	$d_{{Cu_{2} o}} ,d_{{TiO_{2} }}$
	*V*_*OC*_	$d_{{Cu_{2} o}} ,d_{{TiO_{2} }}$	$d_{{Cu_{2} o}} ,d_{{TiO_{2} }}$	$d_{{Cu_{2} o}} ,d_{{TiO_{2} }}$
	*IQE*	$d_{{Cu_{2} o}} ,d_{{TiO_{2} }}$	$d_{{Cu_{2} o}}$	$d_{{Cu_{2} o}} ,d_{{TiO_{2} }}$
$TiO_{2} |Cu_{2} O$ (Ag|Cu)	*J*_*SC*_	$d_{{Cu_{2} o}} ,d_{{TiO_{2} }}$	$d_{{Cu_{2} o}}$	$d_{{Cu_{2} o}}$, *Ratio*
	*V*_*OC*_	$d_{{Cu_{2} o}} ,d_{{TiO_{2} }}$	$d_{{Cu_{2} o}} ,d_{{TiO_{2} }}$	$d_{{Cu_{2} o}}$, *Ratio*
	*IQE*	$d_{{Cu_{2} o}} ,d_{{TiO_{2} }}$	$d_{{Cu_{2} o}}$	$d_{{Cu_{2} o}}$, *Ratio*

### RANSAC derived virtual cells

RANSAC derived models could be used to predict PV properties of virtual solar cell libraries. These predictions could serve two purposes: (1) identify trends related to the dependence of PV properties on descriptors values, which are not easily discernible from the resulting equations. (2) Provide a theoretical basis for and guide future experiments.

#### $TiO_{2} |Cu_{2} O$ (Ag) and $TiO_{2} |Cu_{2} O$ (Ag|Cu) virtual libraries

**Fig. 7:**
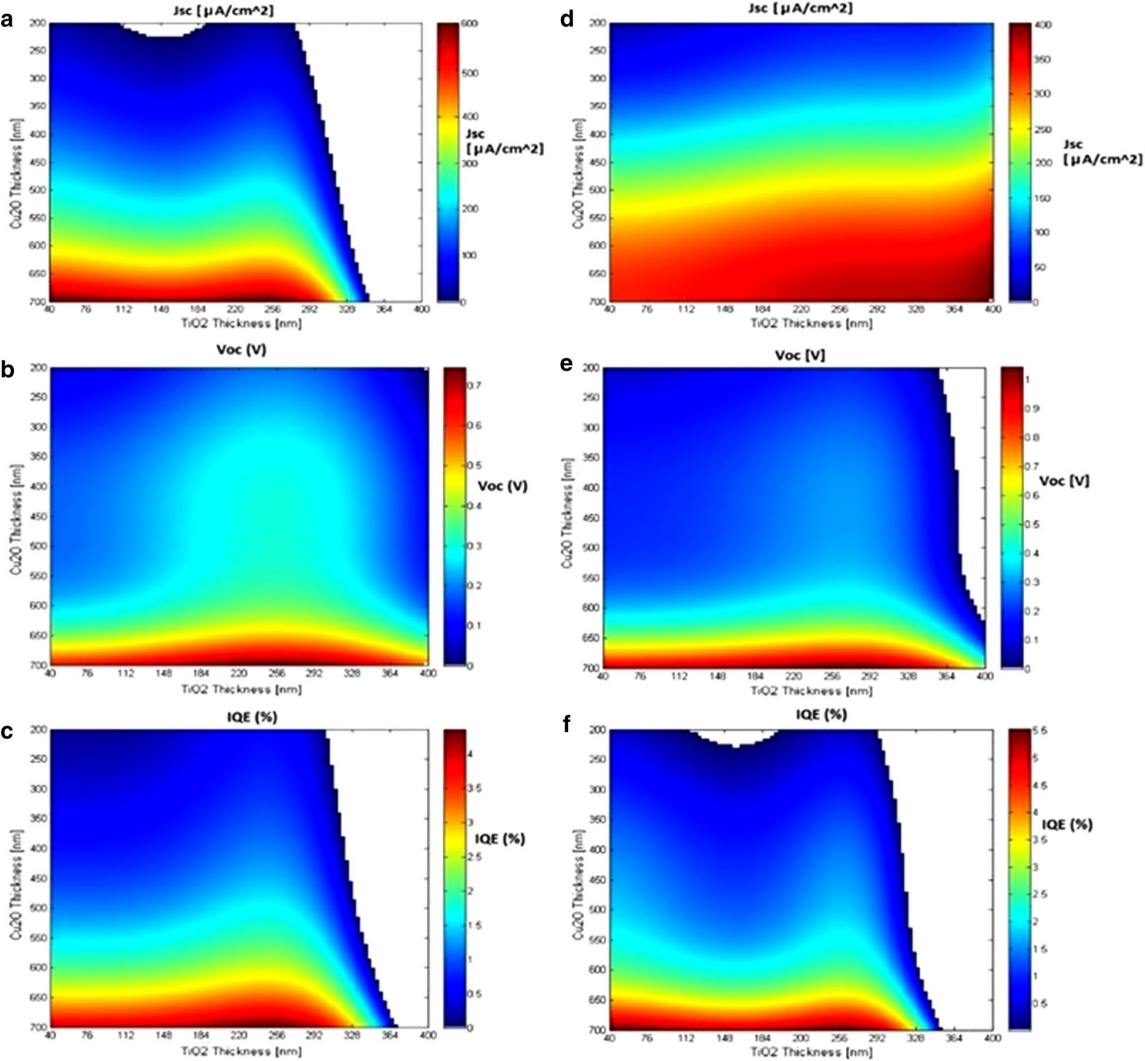
Virtual cells based on the $TiO_{2} |Cu_{2} O$ with Ag back contacts [**a** *J*_*SC*_ (μA/cm^2^); **b** *V*_*OC*_ (V); **c** IQE (%)] and $TiO_{2} |Cu_{2} O$ With Ag|Cu Back Contacts [**d** *J*_*SC*_ (μA/cm^2^); **e** *V*_*OC*_ (V); **f** IQE (%)] solar cells libraries. The *white regions* are outside of the models’ applicability domain

The original $TiO_{2} |Cu_{2} O$ (Ag) and $TiO_{2} |Cu_{2} O$ (*Ag|Cu*) libraries were of identical compositions with $d_{{TiO_{2} }}$ between 70 and 311.5 nm and $d_{{Cu_{2} O}}$ between 249 and 596 nm. The virtual cell should cover these ranges and expand upon them to allow RANSAC-based extrapolations. With this in mind, thickness values for the different layers were selected to be between 200 and 700 nm and between 40 and 400 nm for the Cu_2_O and TiO_2_ layers, respectively, where each range was divided into 100 bins (a total of 10,000 cells per virtual library). These specific ranges were selected following several iterations designed to find the model’s limits, beyond which the results would not be physically meaningful (i.e., have negative PV values). Next, the PV properties (*J*_*SC*_, *V*_*OC*_, *IQE*) of each cell were predicted using the RANSAC models presented in Table 8. The results of these predictions are presented in Fig. 7 and demonstrate a few trends: (1) all PV activities primarily depend on the thickness of the Cu_2_O layer rather than on the thickness of the TiO_2_ layer. This trend was noted by Pavan et al. [43]. but only for *J*_*SC*_. (2) *J*_*SC*_ presents a marked increase for Cu_2_O thicknesses above 500 nm (where *J*_*SC*_ equals $200\frac{\mu A}{{cm^{2} }}$) as seen in Fig. 7a, d. Similar trends (yet with less sharp transitions) are also seen for *IQE* and *V*_*OC*_ (Fig. 7b, e and c, f, respectively). Interestingly, Cu_2_O thicknesses above 500 nm where hardly explored by the original library. (3) The nature of the back contact (Ag vs. Ag|Cu) has the largest effect on the dependence of *J*_*SC*_ on the thickness of the Cu_2_O layer (compare Fig. 7a, d) which is followed by *V*_*OC*_ (compare Fig. 7b, e). In contrast, the dependence of IQE on the thickness of the Cu_2_O layer is the least affected by the back contact (compare Fig. 7c, f). (4) Certain combinations of $d_{{TiO_{2} }}$ and $d_{{Cu_{2} O}}$ are predicted to have both high *J*_*SC*_ and *V*_*OC*_ values. These trends are largely in accord with previous conclusions on these systems deduced from experiments and other data mining approaches [41].

#### $TiO_{2} \left| {Co_{3} O_{4} } \right|MoO_{3}$ virtual library

In a similar manner, another virtual library was constructed for the $TiO_{2} \left| {Co_{3} O_{4} } \right|MoO_{3}$ MOs composition. In the original library, the thicknesses of the different layers ranged from 259 to 355, nm, from 30.7 to 245 nm and from 38.9 to 61.8 for TiO_2_, Co_3_O_4_ and MoO_3_, respectively. In the virtual library, these ranges were increased to 30–500 and 40–100 nm for the Co_3_O_4_ layer and MoO_3_ layer, respectively (50 bins for each range) while the TiO_2_ layer was kept at a constant value of 340 nm. This led to a virtual library consisting of 2500 cells.

**Fig. 8:**
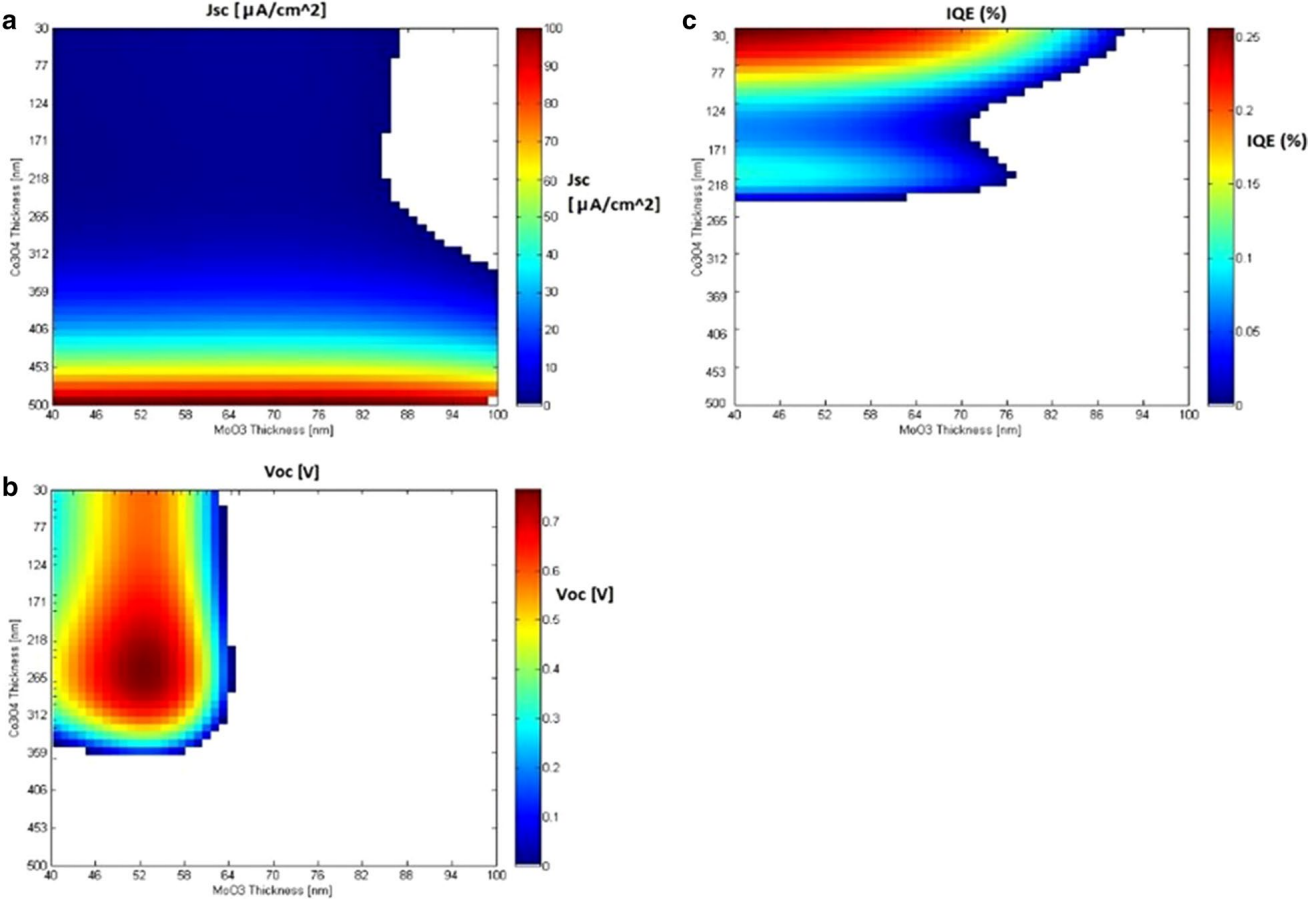
Virtual cells based on the $TiO_{2} \left| {Co_{3} O_{4} } \right|MoO_{3}$ library [**a** *J*_*SC*_ (μA/cm^2^); **b** *V*_*OC*_ (V); **c** IQE (%)]. The *white regions* are outside of the models’ applicability domain

For this particular library, Koushik et al. [15] showed that IQE is mainly affected by the thickness of both the $Co_{3} O_{4}$ and $MoO_{3}$ layers. This conclusion was further supported by a computational analysis [36]. Figure 8 shows that RANSAC’s prediction is in line with this proposition (i.e., to achieve relatively high IQE values, the thickness of the $Co_{3} O_{4}$ layer must be low, smaller than 150 nm and this property is also influenced by the thickness of the $MoO_{3}$ layer). In addition, RANSAC’s models point to an inherent problem in producing solar cells with both high *J*_*SC*_ and IQE values for this MOs combination since the former seems to yield maximum value at $Co_{3} O_{4}$ layer thickness at the 500 nm region while the latter, yields its global maxima at the 30 nm region. Finally, Fig. 8 suggests possible combinations for additional experiments that may lead to high IQE values, for example small thicknesses of both $Co_{3} O_{4}$ and $MoO_{3}$ layers.

## Conclusions

To the best of our knowledge, this is the first application of the RANSAC algorithm in materials-informatics and certainly for the analysis of solar cells libraries. Overall, RANSAC demonstrated a promising ability to develop predictive models for key PV properties across multiple libraries. The statistical parameters of the resulting models favorably compare with results obtained from genetic programing and *k*NN-derived models. Furthermore, the trends observed either from the models in their equation form or from the virtual cells are in agreement with previous findings [43, 45].

The performances of RANSAC together with the ability to use it as a “one stop shop” for model derivation and validation makes the algorithm an appealing additional to the arsenal of modeling tools in chemo- and material-informatics. This opens new opportunities for understanding the factors controlling the properties of materials and for the design of new materials with improved properties. Clearly, the applications of RANSAC (as well as of all other data mining tools) should be conducted in close collaboration with experimentalists to provide physics/chemistry based explanation to the observed trends and to capitalize on the results. We expect that the RANSAC algorithm will find multiple usages in chemoinformatics and materials-informatics researches.
